# Proinflammatory Cytokines (IL-1*α*, IL-6) and Hepatocyte Growth Factor in Patients with Alcoholic Liver Cirrhosis

**DOI:** 10.1155/2015/532615

**Published:** 2015-08-20

**Authors:** Andrzej Prystupa, Paweł Kiciński, Jarosław Sak, Anna Boguszewska-Czubara, Anna Toruń-Jurkowska, Wojciech Załuska

**Affiliations:** ^1^Department of Internal Medicine, Medical University of Lublin, Staszica 16, 20-081 Lublin, Poland; ^2^Department of Family Medicine, Medical University of Lublin, Staszica 11, 20-081 Lublin, Poland; ^3^Department of Ethics and Human Philosophy, Medical University of Lublin, Staszica 4/6, 20-059 Lublin, Poland; ^4^Department of Nephrology, Medical University of Lublin, Jaczewskiego 8, 20-954 Lublin, Poland; ^5^Department of Medical Chemistry, Medical University of Lublin, Chodźki 4a, 20-093 Lublin, Poland; ^6^Department of Mathematics and Medical Biostatistics, Medical University of Lublin, Jaczewskiego 4, 20-954 Lublin, Poland

## Abstract

*Background*. The aim of the study was to assess the activity of interleukin-1*α*, interleukin-6, and hepatocyte growth factor protein (HGF) in serum of patients with alcoholic liver cirrhosis. *Materials and Methods*. Sixty patients with alcoholic liver cirrhosis treated in various hospitals were randomly enrolled. The stage of cirrhosis was assessed according to the Child-Turcotte-Pugh scoring system. The control group consisted of ten healthy persons without liver disease, who did not drink alcohol. Additionally, the group of alcoholics without liver cirrhosis was included in the study. The activity of interleukin-1*α*, interleukin-6, and HGF in blood plasma of patients and controls was measured using the sandwich enzyme immunoassay technique with commercially available quantitative ELISA test kits. *Results*. Higher concentrations of HGF protein were demonstrated in patients with Child class B and Child class C liver cirrhosis, compared to controls and alcoholics without liver cirrhosis. Moreover, significantly higher concentrations of HGF protein were found in patients with Child class C liver cirrhosis compared to patients with Child class A liver cirrhosis (*p* < 0.05). The concentrations of interleukin-1*α* in patients with Child class B and Child class C liver cirrhosis were significantly higher in comparison with controls. Significantly higher concentrations of interleukin-6 were demonstrated in Child class C, compared to Child class A.

## 1. Introduction

Alcoholism is a global health problem. Liver metabolizes most of the ingested alcohol. The pathogenesis of alcohol liver disease and cirrhosis is not well understood. Cirrhosis is defined as an advanced stage of fibrosis, characterized by the formation of regenerative nodules of liver parenchyma that are separated by and encapsulated in fibrotic septa and associated with angioarchitectural changes. The natural history of liver cirrhosis is characterized by a long asymptomatic stage (compensated cirrhosis) with low mortality, followed by a symptomatic decompensated phase characterised by the clinical consequences of liver failure and portal hypertension and by high mortality. There is evidence supporting an important role of cytokines, including interleukin- (IL-) 1*α*, IL-6, and tumor necrosis factor (TNF) in various aspects of inflammatory liver diseases. These cytokines are produced in the liver by Kupffer cells and hepatocytes, playing roles in hepatic inflammation [1, 2]. Hepatic fibrosis is commonly preceded by chronic inflammation [3], and persistence of this inflammation has been associated with progressive hepatic fibrosis and the development of cirrhosis. Liver inflammation is associated with hepatocyte necrosis and apoptosis.

Hepatocyte growth factor (HGF) protein was originally cloned and identified as a potent growth factor for hepatocytes [4]. HGF protein plays an essential part in the development and regeneration of the liver and shows antiapoptotic activity in hepatocytes [5].

The aim of the study was to evaluate concentrations of HGF protein and proinflammatory cytokines IL-1*α* and IL-6 in serum of patients with different stages of alcoholic liver cirrhosis.

## 2. Materials and Methods

This study has been approved by the local Ethical Committee at Medical University of Lublin, Poland (agreement number KE-0254/190/2011). After written informed consent, 60 patients with alcoholic liver cirrhosis treated in various hospitals of the Lublin region were randomly enrolled. The stages of cirrhosis were assessed according to the Child-Turcotte-Pugh criteria (Pugh-Child score) as P-Ch A, P-Ch B, and P-Ch C. The control group consisted of 10 healthy individuals without liver disease, who did not drink alcohol. Additionally, the group of alcoholics without liver cirrhosis was included in the study. Characteristics of the study population are given in Table 1. Cases and controls were age and gender matched.

The diagnosis of liver cirrhosis was based on clinical features, laboratory tests (Table 2), abdominal ultrasound, and history of heavy alcohol consumption (Table 1).

The blood was sampled; after centrifugation, the serum was collected for further analysis.

The levels of IL-1*α*, IL-6, and HGF in serum of patients and controls were measured employing the quantitative sandwich enzyme immunoassay technique with commercially available quantitative ELISA test kits (Quantikine Elisa Human IL-1*α* and Human IL-6, R&D Systems Europe Ltd., and hepatocyte growth factor, USCN Life Science Inc.). Measurements were conducted according to the manufactures' guidelines on a microplate reader (EPOCH; BioTek Instruments Inc.) at 450 nm with wavelength correction at 540 nm. All samples were measured as duplicates and the mean was calculated for data analysis. A calibration curve and a negative control (a blank well without plasma) were run for each test plate.

The Shapiro-Wilk test was used for normal distribution analysis. The Brown-Forsythe test was used for estimation of equality of variances. To compare the results between dependent variables, HGF, Il-1*α* and IL-6, and the independent variable “group,” which divide the population into 5 categories: healthy, alcoholics without liver cirrhosis (O), and alcoholics with classes A, B, and C liver cirrhosis, the Kruskal-Wallis rank sum test was used, a nonparametric equivalent of ANOVA. The Dunn test was applied for detailed identification of statistically different groups. The Spearman rank correlation coefficient was used for evaluation of the relation between variables with a level of significance at *p* < 0.05.

To analyze the influence of variables on the risk of liver cirrhosis, the group of patients was divided into two subgroups: healthy persons without liver cirrhosis and cirrhotic patients (Pugh-Child stages A–C) and univariate logistic regression was used.

## 3. Results

The analysis of data revealed statistically significant intergroup differences in HGF concentrations (H = 45,266; *p* = 0.000).

**Figure 1:**
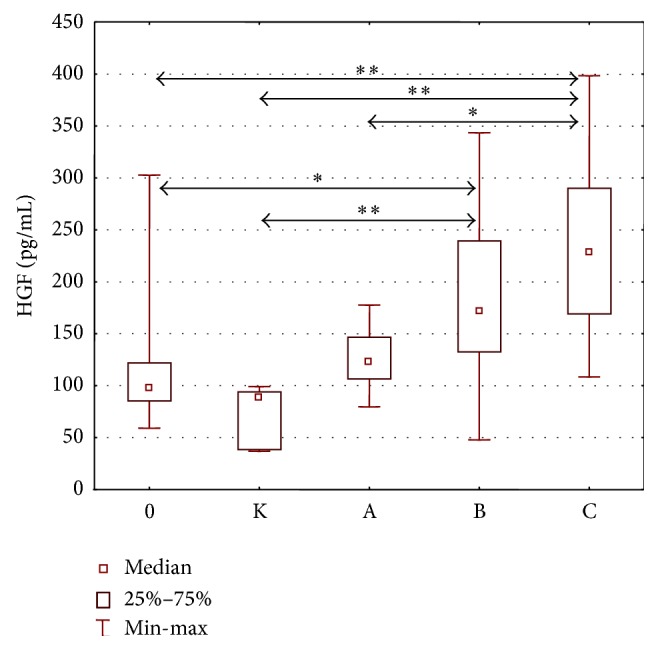
HGF concentration (pg/mL) in the serum of alcoholics without liver cirrhosis (0), alcoholics with liver cirrhosis (stages A, B, and C), and healthy controls (K). Key: ^*∗*^ *p* < 0.05 and ^*∗∗*^ *p* < 0.001.

The detailed analysis demonstrated significantly higher HGF concentrations in patients with Child class B and Child class C liver cirrhosis as compared to the control group and alcoholics without liver cirrhosis (Figure 1). Moreover, significantly higher concentrations of HGF were found in the group of patients with class C liver cirrhosis compared to the group of patients with liver cirrhosis A (*p* < 0.05) (Figure 1). Concentrations of HGF in individual stages of liver cirrhosis are presented in Table 3.

**Figure 2:**
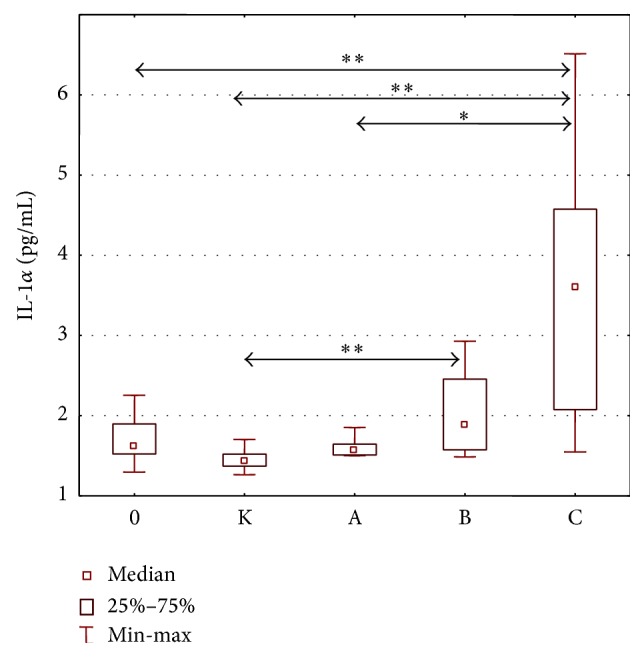
IL-1*α* concentration (pg/mL) in the serum of alcoholics without liver cirrhosis (0), alcoholics with liver cirrhosis (stages A, B, and C), and healthy controls (K). Key: ^*∗*^ *p* < 0.05 and ^*∗∗*^ *p* < 0.001.

Statistically significant intergroup differences in IL-1*α* concentrations were found (H = 50.845; *p* = 0.000). Further detailed analysis revealed significantly higher IL-1*α* concentrations in patients with classes B and C liver cirrhosis in comparison with the control group. Moreover, significantly higher concentrations of IL-1*α* were observed in the group with class C cirrhosis, as compared to alcoholics without cirrhosis and the group with class A liver cirrhosis (Figure 2). The concentrations of IL-1*α* in individual stages of cirrhosis are presented in Table 4.

**Figure 3:**
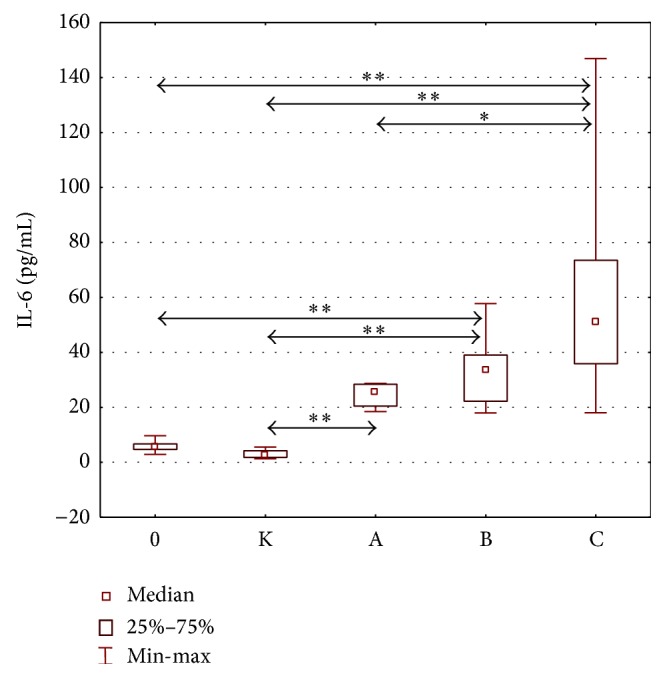
IL-6 concentration (pg/mL) in the serum of alcoholics without liver cirrhosis (0), alcoholics with liver cirrhosis (stages A, B, and C), and healthy controls (K). Key: ^*∗*^ *p* < 0.05 and ^*∗∗*^ *p* < 0.001.

Statistically significant intergroup differences in IL-6 concentrations were demonstrated (H = 69.098; *p* = 0.000). The concentrations of IL-6 in the group with classes A, B, and C liver cirrhosis were significantly higher than those in the control group. Moreover, significantly higher IL-6 concentrations were observed in the group with classes B and C liver cirrhosis, as compared to the group of alcoholics without cirrhosis (Figure 3). Higher IL-6 concentrations were also found in the group with class C liver cirrhosis in comparison with the group with class A cirrhosis. The concentrations of IL-6 in individual stages of liver cirrhosis are presented in Table 5.

**Figure 4:**
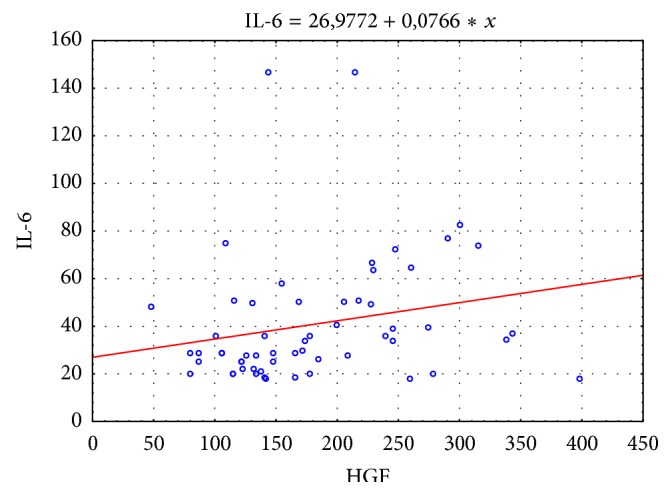
Correlation between HGF and IL-6 in the study population (*r* = 0.32; *p* = 0.016).

**Figure 5:**
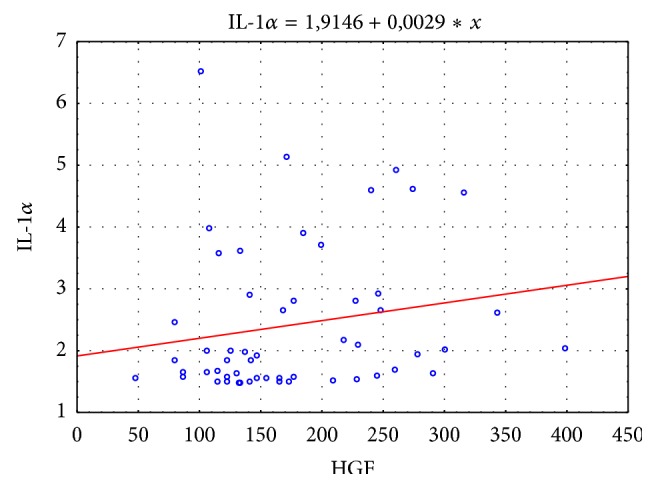
Correlation between HGF and IL-1*α* in the study population (*r* = 0.26; *p* = 0.048).

A statistically significant positive correlation was found between concentrations of HGF versus concentrations of IL-1*α* and IL-6 in the group of patients with liver cirrhosis. The concentration of HGF increases with an increase in IL-6 and IL-1*α* concentrations (Figures 4 and 5).

**Figure 6:**
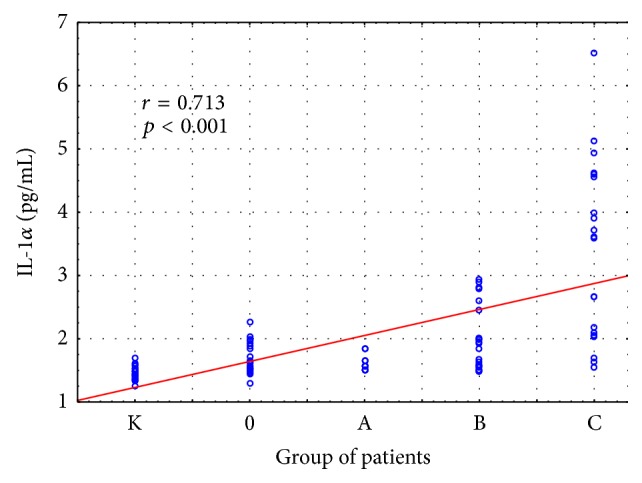
Relationships between IL-1*α* and stage of liver cirrhosis. Key: 0: alcoholics without liver cirrhosis; A, B, and C: alcoholics with liver cirrhosis Pugh-Child stages A–C; K: healthy controls.

**Figure 7 fig7:**
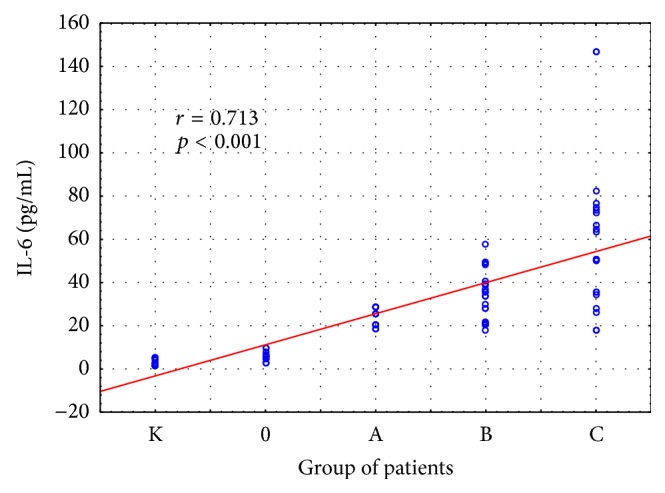
Relationships between IL-6 and stage of liver cirrhosis. Key: 0: alcoholics without liver cirrhosis; A, B, and C: alcoholics with liver cirrhosis Pugh-Child stages A–C; K: healthy controls.

**Figure 8:**
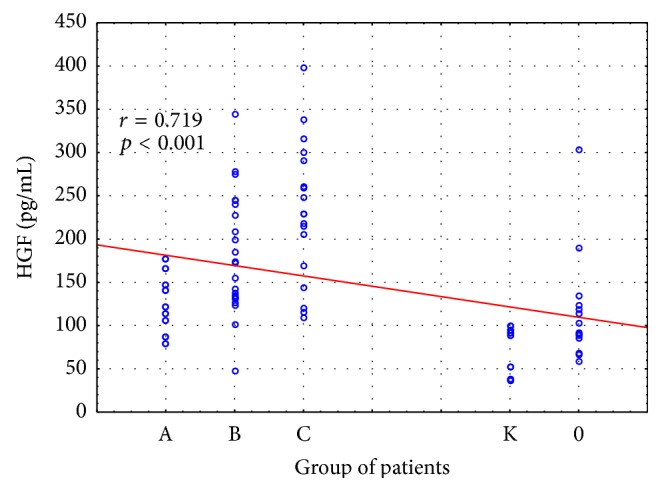
Relationships between HGF and stage of liver cirrhosis. Key: 0: alcoholics without liver cirrhosis; A, B, and C: alcoholics with liver cirrhosis Pugh-Child stages A–C; K: healthy controls.

The relationships between HGF and proinflammatory cytokines and stage of liver cirrhosis were also analyzed. There was significant positive correlation between HGF, IL6, IL-1*α*, and stage of liver cirrhosis (*p* < 0.001) (Figures 6 [Fig fig7]–8).

The univariate logistic regression analysis showed that levels of IL-1*α*, IL-6, and HGF had influenced liver cirrhosis risk (*p* < 0.001) (Table 6).

An increase of one unit (1 pg/mL) of the IL-1*α* concentration resulted in 8% increased risk of the liver cirrhosis, an increase of one unit (1 pg/mL) of IL-6 concentration implicated about 5% increased risk, and an increase of one unit (1 pg/mL) of HGF implicated 3% increased risk of liver cirrhosis.

## 4. Discussion

In the study, concentrations of HGF, IL-1*α*, and IL-6 were analyzed in patients with alcoholic liver cirrhosis classified according to the Child-Pugh scoring system. We showed that the concentration of HGF increases with the progression of liver cirrhosis. The highest HGF concentrations were observed in patients with class C alcoholic liver cirrhosis. Moreover, a correlation between HGF concentration and concentrations of IL-1*α* and IL-6 was found. The concentration of HGF increases with an increase in IL-1*α* and IL-6 concentrations. It is known that, by stimulating hepatocyte proliferation, hepatocyte growth factor has important effects on repair [6]. Plasma HGF concentrations are higher not only in liver diseases but also in patients with acute infections (positively correlating with inflammatory parameters, including C-reactive protein) [7]. In liver diseases, serum levels of HGF can increase due to enhanced production and decreased hepatic clearance, as the liver is the major organ through which HGF is eliminated from circulation.

HGF is produced not only by the liver, but also by the spleen and bone marrow. In liver cirrhosis, the function of the spleen is deteriorating and serum HGF protein levels can be elevated in these patients due to overproduction of HGF protein by the spleen. HGF protein is also produced in the lungs and kidney, the organs involved in liver regeneration [8].

Serum HGF protein levels in Child class C were significantly higher than those in Child class A or B, indicating that these levels can indicate the severity of liver dysfunction in liver cirrhosis [9].

The studies performed in patients with chronic hepatitis C have demonstrated that higher HGF concentrations were associated with increased fibrosis [10] and angiogenesis [11] and have indicated a higher risk of development of hepatocellular cancer [12, 13].

There are only few reports on HGF in patients with alcoholic liver disease and the majority of them were based on small groups of patients. Taïeb et al. have demonstrated significantly higher plasma concentrations of HGF and HGF released by neutrophils in the group of patients with alcoholic hepatitis, as compared to the control group [14]. According to Antoljak et al. and Mendenhall et al. [15, 16], the concentrations of HGF are higher in patients with alcoholic liver cirrhosis, as compared to healthy individuals. As in our study, a correlation has been found between the concentration of HGF and stage of cirrhosis. Moreover, Mendenhall et al. have demonstrated that low plasma concentration of HGF is a factor associated with good prognosis and substantially longer survival of patients.

It was shown by Malatino et al. that increased HGF concentrations are related to an increase in inflammatory process, thickening of the intima-media complex in carotid arteries, and more importantly poor prognosis regarding survival of patients with end-stage renal failure [17]. Noteworthy, results of clinical studies are inconsistent with experimental data showing positive HGF effects on in vitro hepatocyte survival [18]. Hepatocyte growth factor/scatter factor (HGF/SF) has been shown to play an important role in tumor migration and metastasis [19]. IL-6 has been considered to exert a profibrogenic and mitoinhibitory influence on the development of cirrhosis [20]. In patients with liver cirrhosis, however, acute phase response is impaired although systemic IL-6 is markedly increased [21].

Patients with alcoholic liver cirrhosis had higher IL-6 in all blood compartments than patients with cryptogenic liver cirrhosis. Increased systemic IL-6 in patients with liver cirrhosis can be explained by an impaired hepatic removal of IL-6. IL-6 exerts hepatoprotective effects and helps to maintain liver mass [22]. Improvement of hepatic IL-6 removal and signaling may partly ameliorate liver function of patients with liver cirrhosis [23].

IL-1*α* is a multifunctional cytokine and enhances collagen synthesis by increasing the proliferative activity of perisinusoidal cells and has been implicated in the pathogenesis of hepatic fibrosis [2]. Serum levels of interleukin-1*α*, interleukin-6, and C-reactive protein were investigated in patients with chronic liver diseases (CLD). Elevated concentrations of cytokines represent a characteristic feature of CLD, regardless of underlying disease. Enhanced endogenous cytokine levels represent a consequence of liver dysfunction rather than of inflammatory disease [24]. Diez Ruiz et al. found that serum concentrations of interleukin-1*β* and interleukin-6 were significantly raised in alcoholic cirrhosis patients but without any significant differences between patients with liver disease of different grades of severity [25]. In this study, elevation of serum Il-1*α*, IL6, and HGF levels correlated with the progression of liver cirrhosis.

According to our best knowledge, relationship between serum concentrations of HGF and serum concentrations of the proinflammatory cytokines (Il-1*α*, Il-6) in patients with alcoholic cirrhosis has not been described yet.

## 5. Conclusions

Concentrations of HGF, IL-1*α*, and IL-6 increase with severity of alcoholic liver cirrhosis, and the concentration of HGF increases with an increase in proinflammatory cytokines concentration. The concentration of HGF can be a marker of severity of alcoholic liver cirrhosis.

## Figures and Tables

**Table 1:** Characteristic of alcoholics (P-Ch stages 0, A, B, and C) and healthy controls (C).

	C (*n* = 10)	P-Ch 0 (*n* = 22)	P-Ch A (*n* = 14)	P-Ch B (*n* = 25)	P-Ch C (*n* = 24)
Sex (male/female)	8/2	18/4	11/3	20/5	19/5
Age (years)	55.51 ± 8.89	54.91 ± 12.82	52.50 ± 16.11	54.00 ± 12.19	50.71 ± 10.00
Body weight (kg)	75.63 ± 9.83	64.54 ± 8.58	66.33 ± 11.93	84.84 ± 27.11	85.91 ± 21.76
Height (cm)	173.54 ± 10.31	169.91 ± 6.95	171.33 ± 9.86	177.36 ± 11.40	175.45 ± 6.69
Drinking period (years)	—	7.5 ± 2.89	11.16 ± 7.403	13.86 ± 7.06	18.17 ± 10.73

Existing medical symptoms					
Ascites	0	0	0	14	22
Encephalopathy	0	0	1	8	17
Oesophageal varices	0	0	0	9	16

Key: data are expressed as mean ± SD. P-Ch 0: alcoholics without liver cirrhosis; P-Ch stages A, B, and C: alcoholics with liver cirrhosis Pugh-Child stages A–C; C: healthy controls.

**Table 2:** Biochemical data of the study participants.

	C (*n* = 10)	P-Ch 0 (*n* = 22)	P-Ch A (*n* = 14)	P-Ch B (*n* = 25)	P-Ch C (*n* = 24)
Bilirubin (mg/dL)	0.64 ± 0.22	2.66 ± 0.82	2.7 ± 0.95	5.58 ± 0.82	9.71 ± 0.98
Albumin (g/dL)	5.23 ± 0.54	4.20 ± 0.74	4.00 ± 0.67	3.80 ± 0.84	2.42 ± 0.48
ALT (U/L)	19.24 ± 8.56	34.10 ± 8.21	56.63 ± 15.51	63.19 ± 10.38	70.31 ± 18.22
AST (IU/L)	17.81 ± 5.030	42.51 ± 26.45	53.50 ± 27.36	152.9 ± 114.3	190.2 ± 255.1
AST/ALT ratio	0.96 ± 0.21	1.96 ± 1.07	2.67 ± 2.22	2.83 ± 1.35	3.39 ± 1.73
GGTP (IU/L)	20.40 ± 8.96	234.81 ± 46.95	313.75 ± 27.96	642.24 ± 70.04	749.48 ± 72.55
Urea (mg/dL)	24.40 ± 10.07	35.45 ± 8.62	38.77 ± 6.98	44.81 ± 8.54	51.25 ± 5.39
Blood platelets (K/*μ*L)	340.2 ± 7.96	320.95 ± 6.46	166.75 ± 11.96	135.46 ± 12.28	105.33 ± 7.02
INR	1.26 ± 0.16	1.24 ± 0.16	1.30 ± 0.21	1.39 ± 0.23	2.01 ± 0.90
MCV (fl)	86.00 ± 7.26	97.31 ± 7.24	95.97 ± 9.36	97.09 ± 6.27	103.07 ± 6.09
Na (mmol/L)	139.50 ± 3.44	133.56 ± 4.77	129.75 ± 10.50	134.05 ± 4.78	131.85 ± 8.41
K (mmol/L)	4.17 ± 0.32	4.02 ± 0.70	3.59 ± 0.42	4.07 ± 0.77	3.86 ± 0.60

Key: data are expressed as mean ± SD.

P-Ch 0: alcoholics without liver cirrhosis; P-Ch stages A, B, and C: alcoholics with liver cirrhosis Pugh-Child stages A–C; C: healthy controls.

Normal range: bilirubin (0–1.2 mg/dL), albumin (3.5–5.20 g/dL), ALT (alanine aminotransferase) (5–40 U/L), AST (aspartate aminotransferase) (5–40 IU/L), GGTP (Gamma-glutamyl transpeptidase) (11–50 IU/L), urea (21–43 mg/dL), blood platelets (120–400 K/*μ*L), INR (0.86–1.30), MCV (mean cell volume) (80–94 fl), Na (sodium) (136–145 mmol/L), and K (potassium) (3.5–5.1 mmol/L).

**Table 3:** HGF concentration (pg/mL) in the serum of alcoholics and healthy controls.

Groups	Mean ± SD	Median	Minimum	Maximum
C	71.34 ± 26.74	88.12	36.91	99.07
P-Ch 0	116.73 ± 62.94	97.37	59.17	302.61
P-Ch A	126.67 ± 32.79	122.19	79.46	177.40
P-Ch B	180.51 ± 68.74	171.58	47.69	343.53
P-Ch C	231.26 ± 80.51	228.85	108.28	398.34

Key: P-Ch 0: alcoholics without liver cirrhosis; P-Ch stages A, B, and C: alcoholics with liver cirrhosis Pugh-Child stages A–C; C: healthy controls.

**Table 4:** IL-1*α* concentration (pg/mL) in the serum of alcoholics and healthy controls.

Groups	Mean ± SD	Median	Minimum	Maximum
C	1.46 ± 0.11	1.44	1.26	1.70
P-Ch 0	1.69 ± 0.25	1.62	1.30	2.25
P-Ch A	1.61 ± 0.13	1.57	1.50	1.85
P-Ch B	1.99 ± 0.51	1.89	1.49	2.93
P-Ch C	3.38 ± 1.41	3.60	1.55	6.51

Key: P-Ch 0: alcoholics without liver cirrhosis; P-Ch stages A, B, and C: alcoholics with liver cirrhosis Pugh-Child stages A–C; C: healthy controls.

**Table 5:** IL-6 concentration (pg/mL) in the serum of alcoholics and healthy controls.

Groups	Mean ± SD	Median	Minimum	Maximum
C	2.88 ± 1.56	2.33	1.35	5.48
P-Ch 0	5.92 ± 2.04	5.69	2.88	9.64
P-Ch A	24.45 ± 4.09	25.33	18.52	28.76
P-Ch B	33.72 ± 11.25	33.66	18.00	57.77
P-Ch C	60.34 ± 34.94	50.99	18.04	146.92

Key: P-Ch 0: alcoholics without liver cirrhosis; P-Ch stages A, B, and C: alcoholics with liver cirrhosis Pugh-Child stages A–C; C: healthy controls.

**Table 6:** Univariate logistic regression analysis of the risk of liver cirrhosis depending on the level of IL-1*α*, Il-6, and HGF.

*N* = 94	*p*	OR	−95% CI	+95% CI
IL-1*α*	<0.001	1.084	1.046	1.129
Il-6	<0.001	1.05	1.04	1.06
HGF	<0.001	1.0283	1.013	1.043

Key: OR: odds ratio and CI: confidence interval.
